# A Case of Endobronchial Lipoma

**DOI:** 10.1155/DTE.5.263

**Published:** 1999

**Authors:** Hiroshi Iwabuchi, Tetsuo Kamura, Michio Tanaka, Harubumi Kato

**Affiliations:** 1 Department of Surgery Tokyo Metropolitan Hiroo General Hospital 2-34-10 Ebisu, Shibuya-ku Tokyo 150-0013 Japan; 2 Department of Pathology Tokyo Metropolitan Hiroo General Hospital 2-34-10 Ebisu, Shibuya-ku Tokyo 150-0013 Japan; 3 Department of Surgery Tokyo Medical University 6-7-1 Nishishinjuku Shinjuku-ku Tokyo 160-0023 Japan

## Abstract

Endobronchial lipomas are very rare tumors originating from the tracheo-bronchial wall. We have experienced a case of endobronchial lipoma which was found at the orifice of the left lower lobe bronchus and obstructive pneumonia distal to it. Left lower lobectomy was performed, and the patient has been well without any symptoms so far.

Endobronchial lipomas are histologically benign tumors, but more than half of the reported cases underwent lobectomy or pneumonectomy due to the presence of varying degrees of irreversible peripheral lung damages.


*Diagnostic and Therapeutic Endoscopy*, Vol. 5, pp. 263–267
Reprints available directly from the publisher
Photocopying permitted by license only

© 1999 OPA (Overseas Publishers Association) N.V.
Published by license under
the Harwood Academic Publishers imprint,
part of The Gordon and Breach Publishing Group.
Printed in Malaysia.

### A Case of Endobronchial Lipoma

HIROSHI IWABUCHI[a,*], TETSUO KAMURA[a], MICHIO TANAKA[b] and HARUBUMI KATO[c]

[a]*Department of Surgery,* [b]*Department of Pathology, Tokyo Metropolitan Hiroo General Hospital, 2-34-10, Ebisu, Shibuya-ku, Tokyo 150-0013, Japan;* [c]*Department of Surgery, Tokyo Medical University, 6-7-1, Nishishinjuku Shinjuku-ku, Tokyo 160-0023, Japan*

*(Received 14 December 1998; Revised 9 February 1999; In final form 25 February 1999)*

**Endobronchial lipomas are very rare tumors originating from the tracheo-bronchial wall. We have experienced a case of endobronchial lipoma which was found at the orifice of the left lower lobe bronchus and obstructive pneumonia distal to it. Left lower lobectomy was performed, and the patient has been well without any symptoms so far.**

**Endobronchial lipomas are histologically benign tumors, but more than half of the reported cases underwent lobectomy or pneumonectomy due to the presence of varying degrees of irreversible peripheral lung damages.**

*Keywords:* Bronchial biopsy, Endobronchial lipoma, Obstructive pneumonia, Surgical treatment

### INTRODUCTION

Endobronchial lipomas are very uncommon tumors found in the tracheobronchial tree. They are thought to originate from the fatty tissue normally present in the bronchial wall. Lipomas are histologically benign tumors, but as a result of the intraluminal location, obstructive pneumonia and subsequent irreversible lung damage are often observed. Therefore, many cases have required surgical resection.

In this paper, we present a case in which a left lower lobectomy was performed to treat endobronchial lipoma.

### CASE REPORT

A 62 year-old woman visited another hospital on June 8th, 1998, due to persistent cough and spiking fever. She had had a medical history of recurrent pneumonia several times during the previous 7 years. A chest X-ray film (Fig. 1) was taken immediately and an inflammatory shadow was pointed out in the left lower lung field. Therefore, she was referred to our hospital for more detailed examinations and treatment. A chest CT scan on admission (Fig. 2) revealed a low-density tumor mass at the orifice of the left lower lobe bronchus and obstructive pneumonia distal to it. Both sputum

* Corresponding author.

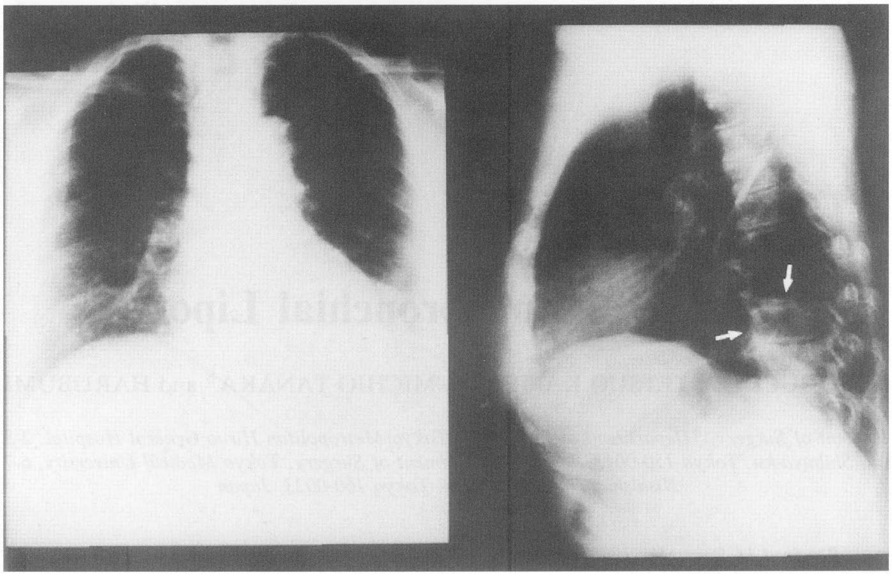

FIGURE 1 Chest X-ray film showed an inflammatory shadow (arrows) in the left lower lung field.

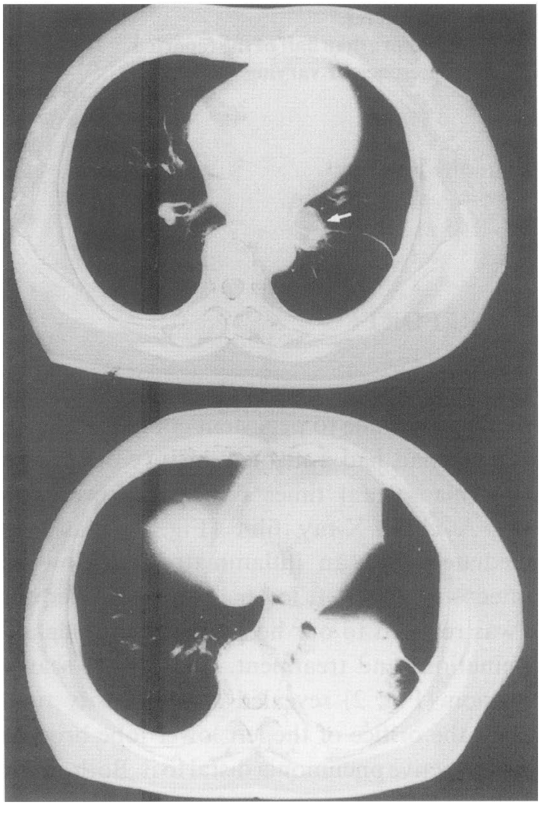

FIGURE 2 Chest CT scan revealed a low-density tumor mass (arrow) at the orifice of the left lower lobe bronchus (upper) and obstructive pneumonia distal to it (lower).

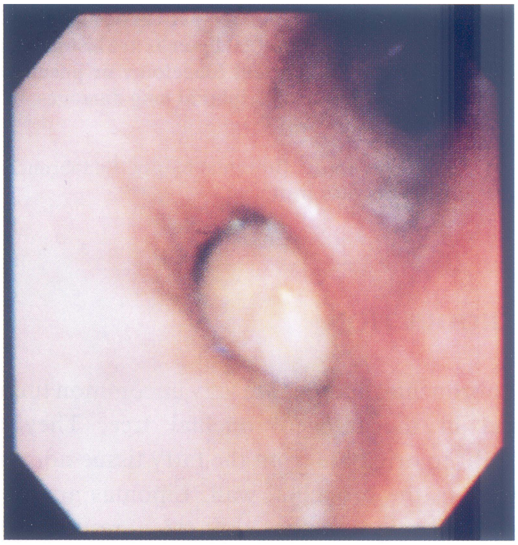

FIGURE 3 Bronchoscopy revealed the left lower lobe bronchus to the almost completely obstructed by a smooth surfaced polypoid tumor.

cytology and culture were negative. Pulmonary function tests were within normal limits. Bronchoscopy revealed the left lower lobe bronchus to be almost completely obstructed by a smooth surfaced polypoid tumor. Figure 3 shows the polypoid tumor at the orifice of the left lower lobe bronchus.

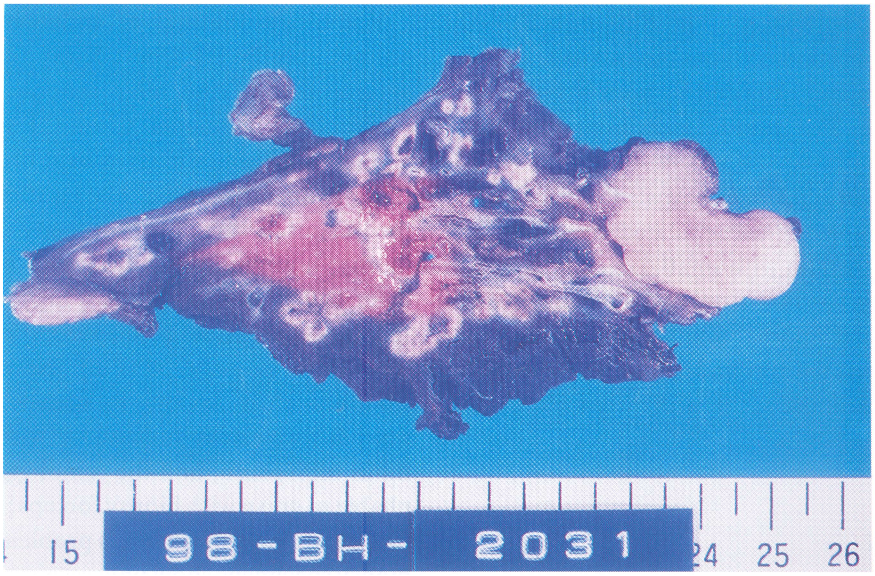

FIGURE 4 Macroscopic findings showed the dumbbell-shaped tumor extended to both sides of the bronchial wall. Peripheral lung was severely damaged by recurrent pneumonia.

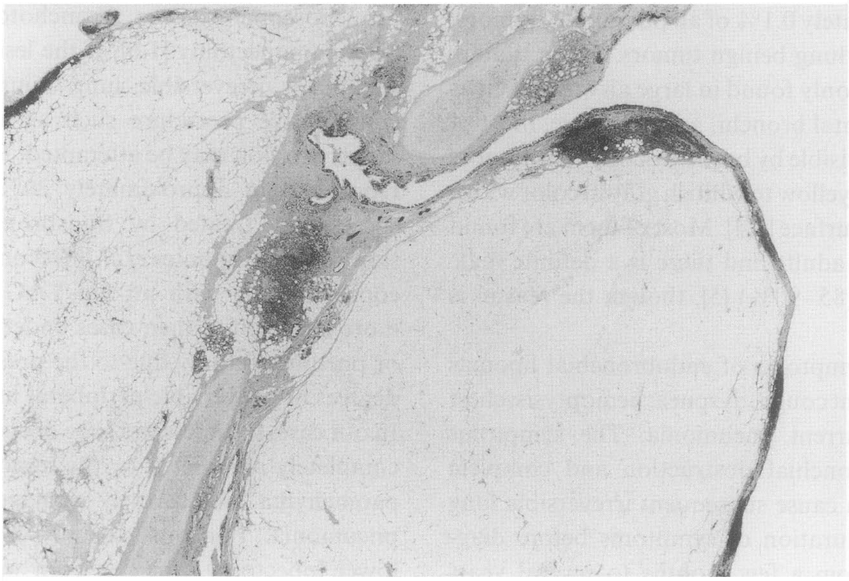

FIGURE 5 Adipose tissue completely encapsulated by columnar respiratory epithelium with small foci of squamous metaplasia.

A bronchial biopsy specimen showed chronic bronchitis with focal squamous metaplasia and a small section of fat tissue, but a definitive diagnosis was not obtained. We evaluated this case and decided that left lower lobectomy was necessary for definitive diagnosis and treatment. Operation was performed on the 27th of July. Severe pleural adhesion and local atelectasis were found in the left lower lobe. The soft, pale-yellow tumor was found at the orifice of left lower bronchus, and lower lobectomy was performed. The dumbbell-shaped tumor (Fig. 4) originated from the cartilage of left lower bronchus and extended to both sides of the bronchial wall. The tumor measured 30 × 22 × 14 mm.

Histological examination revealed mature adipose tissue completely encapsulated by columnar respiratory epithelium with small foci of squamous metaplasia (Fig. 5). The histological diagnosis was an endobronchial lipoma. She was discharged on the 10th postoperative day and has been followed up in our outpatient department, with no complaints or symptoms.

### DISCUSSION

Endobronchial lipomas are very rare tumors originating from the adipose tissue that is normally present in the tracheobronchial wall [1]. Schraufnagel *et al.* [2] reported that the frequency of these tumors to be approximately 0.1% of all pulmonary tumors, and 13% of all lung benign tumors. These tumors are more commonly found in large airways, such as lobar or segmental bronchi, and therefore, most of the tumors are visible by bronchoscopy. The tumors are usually pale yellow to whitish gray in color with a smooth round surface [3,4]. Most of them are found in middle aged adults and there is a definite male predominance (85–90%) [5], though the reason is unknown.

Presenting symptoms of endobronchial lipomas include persistent cough, dyspnea, hemoptysis, chest pain, and recurrent pneumonia. The symptoms result from bronchial obstruction and complete obstruction can cause subsequent irreversible lung damage. The duration of symptoms before diagnosis ranges from a few months to several years because the tumor growth is very slow [6]. In this case, the patient had a medical history of recurrent pneumonia during the previous seven years. Eventually, she complained of productive cough and spiking fever for one month. Bronchoscopy showed the tumor to almost completely obstruct the orifice of the left lower lobe bronchus.

Radiologic findings of endobronchial lipomas are not specific [3]. Chest X-ray films often show enlargement of the hilar shadow, atelectasis or obstructive pneumonia. Chest CT scan may reveal a low density tumor mass in large bronchi, but these findings are not specific. The most reliable methods for diagnosis are bronchoscopy and bronchial biopsy, however, the accuracy of bronchial tissue diagnosis is not particulary high. Ovil *et al.* [5] reported that histologic diagnosis by bronchoscopy was made in only 50% of the cases reported. Endobronchial lipoma is usually covered with normal respiratory mucosa or with foci of squamous metaplasia, and the tumor itself may be too pliable to grasp with biopsy forceps [2,7]. Therefore, bronchial tissue diagnosis is problematic. When the biopsy specimens show only respiratory epithelium, it may be very difficult to exclude the possibility of malignancy. In such cases, resection is necessary for a definitive diagnosis.

The treatment of endobronchial lipoma includes bronchoscopic excision, bronchotomy, lobectomy or pneumonectomy [1,6]. If the lesion is small and significant irreversible lung injury is absent, a conservative procedure such as a transbronchoscopic excision may be attempted. Remigio *et al.* [3] reported that approximately 20% of cases were successfully treated by transbronchoscopic excision. Smirniotopoulos *et al.* [8] recommended endoscopic ablation with an Nd-YAG laser, however more than half of their cases underwent lobectomy or pneumonectomy due to the presence of varying degrees of irreversible peripheral lung damages [9]. In our case, the left lower lobe bronchus was almost completely obstructed by the tumor and the distal parenchyma was severely damaged by recurrent pneumonia. For this reason, we performed left lower lobectomy. She has been well and has had no symptoms since the resection. The prognosis of this tumor is generally excellent, because endobronchial lipomas are benign tumors.

Finally, as many authors have emphasized [3,4,9,10], early detection and early removal of endobronchial lipomas may prevent the need for extensive surgical resection.

***Acknowledgment***

The authors thank Professor J. Patrick Barron of the International Medical Communications Center, Tokyo Medical University, for his review of the manuscript.

***References***

[1] Hurt, R. Benign tumors of the bronchus and trachea. *Ann. R. Coll. Surg. Engl.* 1984; **66**: 22–26.

[2] Schraufnagel, D.E., Morin, J.E. and Wang, N.S. Endobronchial lipoma. *Chest* 1979; **75**: 97–99.

[3] Remigio, P.A. and De La Cruz, M. Endobronchial lipoma. *NY State J. Med.* 1988; **88**: 550–551.

[4] Iannicello, C.M., Shoenut, J.P., Sharma, G.P. *et al.* Endobronchial lipoma: Report of three cases. *Canadian J. Surg.* 1987; **30**: 430–431.

[5] Ovil, Y., Schachner, A., Schujman, E. *et al.* Benign endobronchial lipoma masquerading as recurrent pneumonia. *Eur. J. Respir. Dis.* 1982; **63**: 481–483.

[6] MacArthur, C.G.C., Cheung, D.L.C. and Spiro, S.G. Endobronchial lipoma: A review with four cases. *Br. J. Dis. Chest* 1977; **71**: 93–100.

[7] Spinelli, P., Pizzetti, P., Lo Gullo, C. *et al.* Resection of obstructive bronchial fibrolipoma through the flexible fiberoptic bronchoscope. *Endoscopy* 1982; **14**: 61–63.

[8] Smirniotopoulos, T.T., Quate, L.J., Arabian, A. *et al.* Endoscopic removal of a bronchial lipoma with the neodymium-YAG laser. *Endoscopy* 1986; **18**: 197–198.

[9] Yokozaki, M., Kodama, T., Yokose, T. *et al.* Endobronchial lipoma: A report of three cases. *Jpn. J. Clin. Oncol.* 1996; **26**: 53–57.

[10] Matsuba, T., Matsumoto, K., Fujiki, T. *et al.* A case of endobronchial lipoma with distal organizing pneumonia. *J. Jpn. S. Bronch.* 1992; **14**: 201–205.

